# A novel surgical technique to localize small enteropouch fistula

**DOI:** 10.1186/1471-2490-5-16

**Published:** 2005-11-30

**Authors:** Abbas Basiri, Emadoddin Mo'oudi, Hamed Akhavizadegan, Naser ShakhsSalim

**Affiliations:** 1 Professor of Urology, Urology/Nephrology Research Center, Shaheed Labbafinejad Hospital, Shaheed Beheshti University of Medical Sciences, Tehran, Iran; 2 Resident of Urology, Shaheed Labbafinejad Hospital, Shaheed Beheshti University of Medical Sciences, Tehran, Iran

## Abstract

**Background:**

One of the rare complications of ileal neobladder after radical cystectomy is pouch-to-intestine fistula. There isn't a classic method to intraoperative diagnosis of small fistula.

**Case presentation:**

An entero-pouch fistula was occurred in a patient after radical cystectomy with illeal orthotopic pouch. Because of failed conservative management, the patient was candidate for surgery. The hidden small fistula in the small intestine was diagnosed by high intraluminal hydrostatic pressure (by intraluminal saline injection).

**Conclusion:**

Intraoperative diagnosis the intestinal opening of a small fistula is very important. At the time of surgery if the fistula tract becomes open (during releasing the adhesions), it may leak in the peritoneum in postoperative period. Intraluminal high pressure is a useful method for intraoperative small hidden intestine opening.

## Background

One of the complications of ileal neobladder after radical cystectomy is pouch-to-intestine fistula [1]. To date cases with enteropouch [2], enteroconduit [3] and ureteroenteric [4] fistulae have been reported. Patients more often have a history of irradiation [2], but there have been cases observed without it too [5]. Conservative management and surgery both have been used to treat such fistulae [5,6]. To our knowledge there is no published report on the surgical technique used to treat enteropouch fistulae. We report a novel intraoperative technique to identify non-visible fistula.

## Case presentation

**Figure 1:**
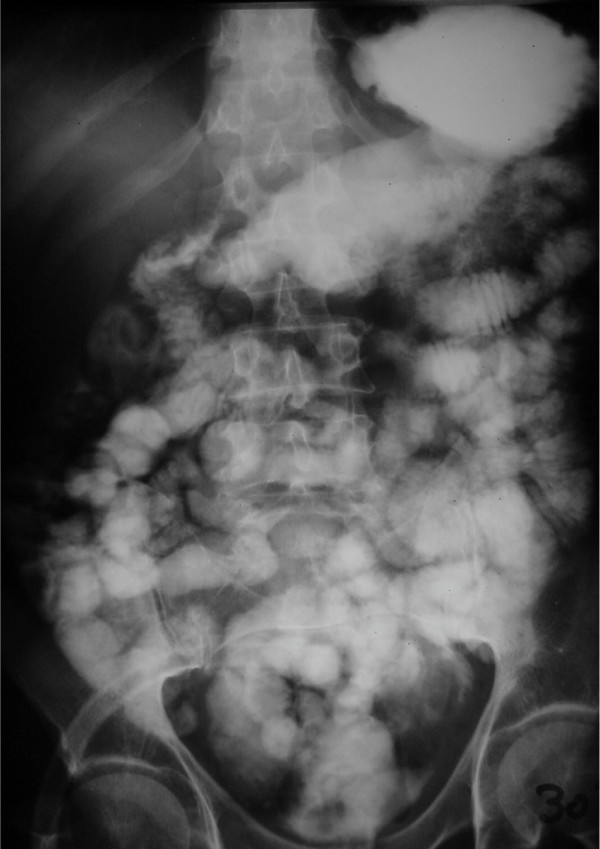
Upper GI series of the patient with entero-pouch fistula.

**Figure 2:**
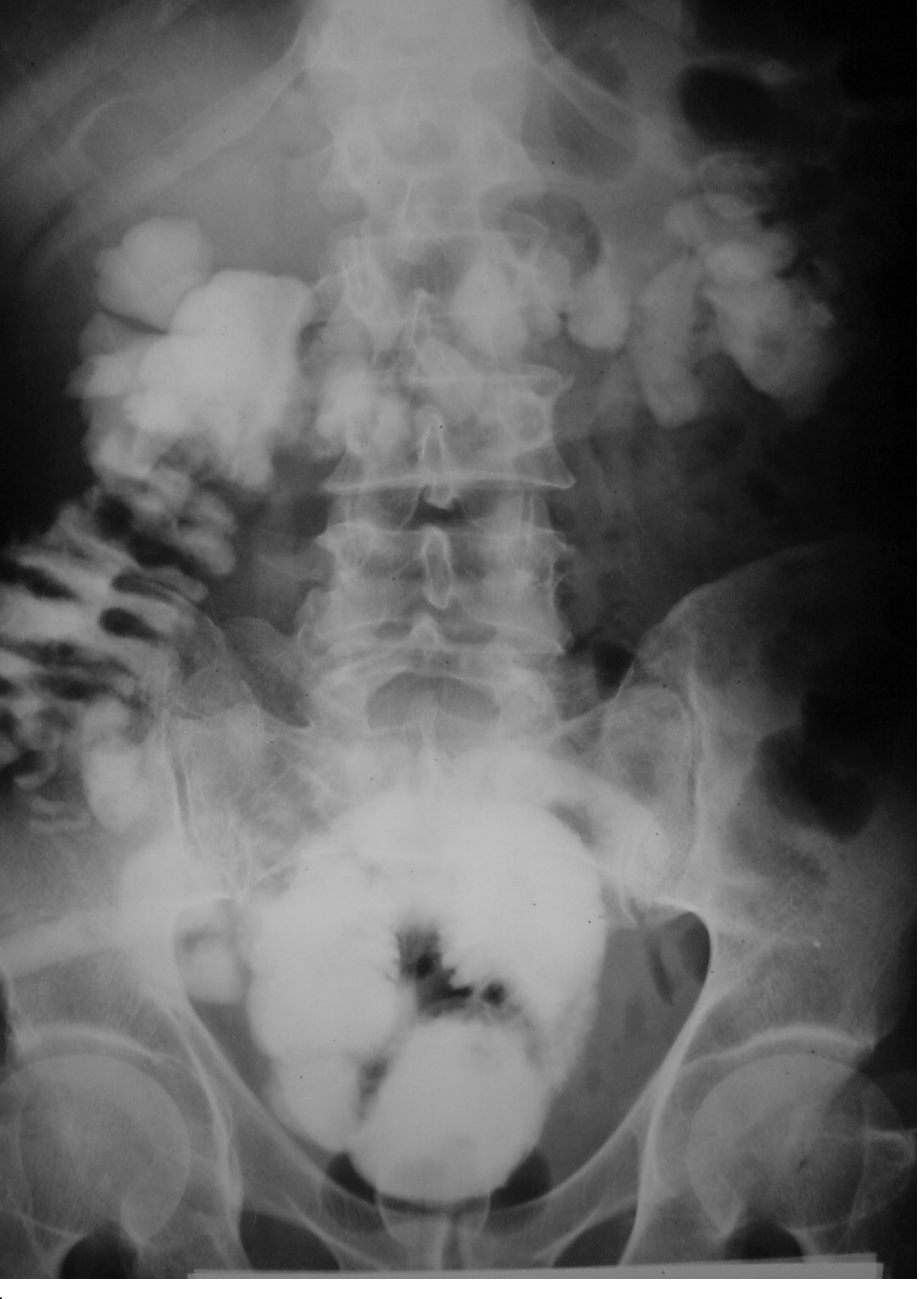
Pouchogram of the patient with entero-pouch fistula.

A 52-year-old male with muscle invasive TCC (grade III) had undergone radical cystectomy and ileal orthotopic pouch reconstruction. Anastomosis of small intestine was performed in two layers (first runing chromic and second with simple separate silk sutures). On the 10^th ^day after the operation while the patient was on normal diet and had no abdominal symptom or defecation disorder, fecaloid materials were found in urine and drained secretions. Enteropouch and entrocutaneous fistulae were confirmed using oral activated charcoal. The patient was put on fasting and TPN. Antibiotic therapy was initiated, consequently, urinary fecaloid drainage and enterocutoneous fistula improved. However enteropouch fistula remained unresolved even after one month of conservative management. The patient was discharged with free urinary drainage and low residue diet for another month, but the fistula persisted. Upper GI series revealed no pertinent information (fig. 1), but pouchogram (fig. 2) revealed that the fistula was proximal to the ileocecal junction and most probably on the bowel anastomosis.

Performing surgery, via previous surgical scar (lower midline) all adhesion bands were released and anastomosis (defined by silk sutures) was separated from the pouch, but no fistula was revealed. Thus, with intestine clamped on each side of anastomosis and saline was injected to the lumen, intraluminal pressure increased gradually and fistula appeared by leaking fluid through it.

Fistula repair was done in two layers (after freshing of its edges) in transverse fashion to decrease the risk of stricture. Omentum was interposed between the pouch and fistula without tension. The patient was on TPN and NPO for 5 days and free drainage for two weeks. After two weeks pouchogram was performed and no fistula was seen subsequently the patient was discharged with clean intermittent catheterization. Follow-up up to one year was indicative of favourable outcome.

## Discussion

Enteropouch fistula is a rare complication after supravesical urinary diversion [5]. Other than surgery, diverticulitis, colorectal malignancy, radiation and Crohn's disease have become the most common aetiology of entero-urinary fistula [7].

In other case reports; fistula was detected three weeks after surgical operation [5,6], while fistula manifestations were observed 10 days after surgery in our patient. According to the published reports, symptoms are watery diarrhea, mild abdominal symptoms (nausea, vomiting, and dehydration, but normal physical examination), gas in the pouch (diagnosed by KUB) and fecaluria [2,6]. In our case the only symptom was fecaluria. All the researchers have mentioned that upper GI series, barium enema, and CT scan are not helpful and the best diagnostic modality is pouchogram [2,6], as it was in our patient. Occasionally, clinical diagnosis in borderline cases is difficult [8]. Some investigators use charchoal [9], methylene blue [8], poppy seed [8], and urine cytology in order to confirm enteropouch fistula diagnosis [10].

The conservative management which is indicated in cases with no sepsis, no obstruction, and no organ impairment and with a normal nutritional status, consists of hyper-alimentation, fasting or low residue diet and continous urinary drainage [7].

Our review results contained cases reported as resistant to conservative management, who were treated surgically [6]. However, the surgical technique used was not elaborated in any of the studies. To our experience, during the operation after releasing all adhesion bands between the pouch and intestinal loops, it's of utmost importance that intestinal opening be defined using intestinal compression in both sides of anastomosis. Otherwise, there is a high probability of inter-loop abscess formation due to the intraperitoneal bowel perforation, which may lead to a high mortality rate.

## Conclusion

Oral administration of activated charcoal is a useful method to help diagnosis of enteropouch fistula. Also, pouchogram is the best diagnostic test.

During the operation, examining the anastomotic site and then all sites of adhesion between intestine and pouch with high intraluminal pressure is an eligible method to detect fistula opening in the intestine.

## Pre-publication history

The pre-publication history for this paper can be accessed here:


